# Summary of the application value of ultrasound imaging features in the clinical differential diagnosis of intramuscular capillary-type hemangioma and fibro-adipose vascular anomaly

**DOI:** 10.3389/fonc.2023.1256667

**Published:** 2023-12-06

**Authors:** Wen-Jia Hu, Hai-Ting Li, Zhi-Na Fan, Yu-Bin Gong, Xiao-Nan Guo, Chang-Xian Dong, Pan-Hong Fan, Xiao Yang, Gang Wu

**Affiliations:** ^1^Department of Ultrasound, Henan Provincial People’s Hospital & Zhengzhou University People’s Hospital & Henan University People’s Hospital, Zhengzhou, China; ^2^Department of Medical imaging, Henan Provincial People’s Hospital & Zhengzhou University People’s Hospital & Henan University People’s Hospital, Zhengzhou, China; ^3^Department of Hemangioma and vascular malformations, Henan Provincial People’s Hospital & Zhengzhou University People’s Hospital & Henan University People’s Hospital, Zhengzhou, China; ^4^Department of Pathology, Henan Provincial People’s Hospital & Zhengzhou University People’s Hospital & Henan University People’s Hospital, Zhengzhou, China; ^5^Department of Ultrasound, Peking Union Medical College Hospital, Chinese Academy of Medical Science & Peking Union Medical College, Beijing, China

**Keywords:** intramuscular capillary-type hemangioma, fibro-adipose vascular anomaly, ultrasound, diagnostic efficacy, application value

## Abstract

**Objective:**

To investigate the value of ultrasonography as a diagnostic aid in differentiating intramuscular capillary-type hemangioma (ICTH) from fibro-adipose vascular anomaly (FAVA).

**Methods:**

A retrospective analysis was conducted of the clinical and ultrasound imaging data of 20 patients with ICTH and 45 patients with FAVA who were admitted to and pathologically confirmed in hospital between January 2013 and April 2023. The clinical and ultrasonographic appearances of the lesions in the two groups were compared and analyzed. A stepwise regression analysis was performed, and a joint diagnostic equation was constructed using the final variables selected. The receiver operating characteristic (ROC) curve and indicators, including sensitivity and specificity, were used to evaluate the efficacy of the joint diagnostic model.

**Results:**

The two groups of patients suffering from ICTH and FAVA presented a statistically significant difference (*P<* 0.05) in terms of ‘age’, ‘lesion size’, ‘fascial tail sign’, ‘presence of a fatty-tissue-like hyperecho around the lesion’, ‘blood flow’ and ‘presence of straight blood capillaries within the lesion’. Finally, the variables ‘fascial tail sign’ and ‘presence of straight blood capillaries within the lesion’ were selected to construct the model. The constructed joint diagnostic model had a sensitivity value of 70.0% (95% CI: 59.00–81.00), a specificity value of 98.0% (95% CI: 94.70–100.00) and a ROC curve value of 0.908, indicating the high efficacy of the combined diagnosis method.

**Conclusions:**

Ultrasonography can be utilized to differentiate ICTH from FAVA, and the combined diagnosis method can further improve the technique’s diagnostic efficacy.

## Introduction

Intramuscular hemangioma is a type of rare benign hemangioma in the skeletal muscles and accounts for about 8% of all hemangiomas (1, 2). In 1972, Allen and Enzinger proposed the first classification of intramuscular hemangiomas and defined the capillary-type hemangioma as an intramuscular hemangioma–capillary type (3). In 2014, Yilmaz et al. officially named it intramuscular capillary-type hemangioma (ICTH) (4), also known as intramuscular hemangioma–capillary type (5) or intramuscular invasive angiolipoma (6). As a rare benign intramuscular hemangioma, in 2018, ICTH was recognized by the International Society for the Study of Vascular Anomalies as a temporarily unclassified vascular disease. Commonly, ICTH is found on the head, neck, trunk and extremities of infants and children and is typically characterized by the presence of a solid mass that grows slowly and does not spontaneously regress in deep soft tissues. It has a low incidence with hided and diverse clinical manifestations.

Currently, due to poor clinical, imaging and pathological knowledge of ICTH, the disease is often misdiagnosed as other pathological types of hemangioma or vascular malformation. It is especially confused with another relatively rare subtype of vascular malformation – the fibro-adipose vascular anomaly (FAVA) (7, 8), which also usually occurs in the muscle – due to the many similarities between them in terms of clinical symptoms and imaging features (9). The clinical features of ICTH include a rich blood supply, pseudo-infiltrative growth (10) and a tendency to relapse, so sclerotherapy is often performed before an operation to prevent excessive blood loss (4). In contrast, although the early symptoms of FAVA, which usually occur in children and adolescents, also include the presence of a painless intramuscular or intermuscular mass, sclerotherapy is not required before surgery. Thus, the preoperative differential diagnosis of the two diseases will help prevent surgical bleeding and reduce surgical difficulty, which may improve the prognosis to some extent.

In this paper, the clinical and imaging data of patients with ICTH/FAVA are analyzed to explore their clinical and ultrasound diagnostic features and identify major differences between the diseases to provide a reference for future clinical practice.

## Materials and methods

### Material selection

Patients pathologically diagnosed with ICTH/FAVA between January 2013 and April 2023 at Henan Provincial People’s Hospital were included. The inclusion criteria were as follows: (1) clinical manifestations and imaging findings were combined, and patients met the pathological diagnostic criteria of ICTH or FAVA; (2) the lesion was located within or between the trunk and limb muscles; (3) complete ultrasound sonogram data were available. The exclusion criteria were as follows: (1) patients who were accompanied by other types of hemangioma or vascular malformation; (2) patients suffering from other organic diseases.

The study was approved by the Ethics Committee of Henan Provincial People’s Hospital. Written informed consent was obtained from the participants or minors’ legal guardians/next of kin for the publication of any potentially identifiable images or data included in this article. Among the 137 patients preliminarily screened, 72 patients were excluded according to the exclusion criteria, and the remaining 65 patients were enrolled, among whom, 20 suffered from ICTH and 45 suffered from FAVA.

### Methods

ACUSON S2000™ and S3000™ (Siemens, Germany) color Doppler ultrasonic diagnosis systems were used for scanning. The probe was placed at the site of the lesion, with a frequency of 6.0–13.0 MHz. The collected images of the patients were obtained by the same in-charge physician from the ultrasound department of the hospital. To obtain optimal images, the depth and gain of the apparatus were adjusted according to the location and size of the lesion. After the lesion was detected, 2D ultrasound imaging was used to transversely and longitudinally measure the lesion’s boundary, envelope, morphology, internal echo, relationship with surrounding tissues and size. In addition, the presence of a ‘fascial tail sign’, namely, a high band type or triangular echo around the lesion, was also examined on the edge of the lesion. The presence of a fatty-tissue-like hyperecho around the lesion was also checked.

For a larger lesion, the panoramic imaging technique was used for full scanning. Colour Doppler ultrasonography was used to observe the blood flow in and around the lesion and whether there were straight blood capillaries. If straight and orderly distributed vessels were observed within the lesion, and no twisted or interlaced vessels were found, the ‘presence of straight blood capillaries’ was concluded. Intra-focal blood flow was quantitatively graded by the Adler semi-quantitative method. Spectral measurements were obtained using pulsed Doppler technology.

### Statistical analysis

A statistical analysis of the data was performed using SPSS 24.0 software. Means and standard deviations were used to statistically describe continuous variables, while *t*-test hypothesis testing was performed to describe variables that met tests for normality and homogeneity of variance. For continuous data that did not meet the parametric test conditions, the rank–sum test was performed; for discrete data, the number of cases and the percentage were used for statistical descriptions, and the Chi-squared test was performed. The data were expressed as $\bar{x}\pm s$ and M (Q1, Q2), respectively. Binary logistic stepwise regression was conducted to analyze the factors that might influence the diagnosis results of both diseases. The receiver operating characteristic (ROC) curve was used to analyze the diagnostic efficiency of the single and combined indicators. The value *P*< 0.05 was considered statistically significant.

## Results

### General data and ultrasound imaging features

Among the 20 patients with ICTH were 9 males and 11 females. The anatomical position of the lesions was as follows: crus (*n* = 5), forearm (*n* = 4), waist/back (*n* = 4), thigh (*n* = 4), thorax and abdomen (*n* = 3). Eight patients had pain and were sensitive to sensitive on palpation, and 12 patients had no symptoms. The mean age was 8.55 ± 8.06 years, and the lesion size was 53.40 ± 26.30 mm.

Among the 45 patients with FAVA were 24 males and 21 females. The anatomical position of the lesions was as follows: crus (*n* = 22), thigh (*n* = 18), forearm (*n* = 2), waist/back (*n* = 2), foot (*n* = 1). Thirty-five patients had pain and were sensitive to pressing, and 10 patients had no symptoms. The mean age was 20.00 ± 13.99 years, and the mean lesion size was 86.98 ± 44.78 mm.

**Figure 1:**
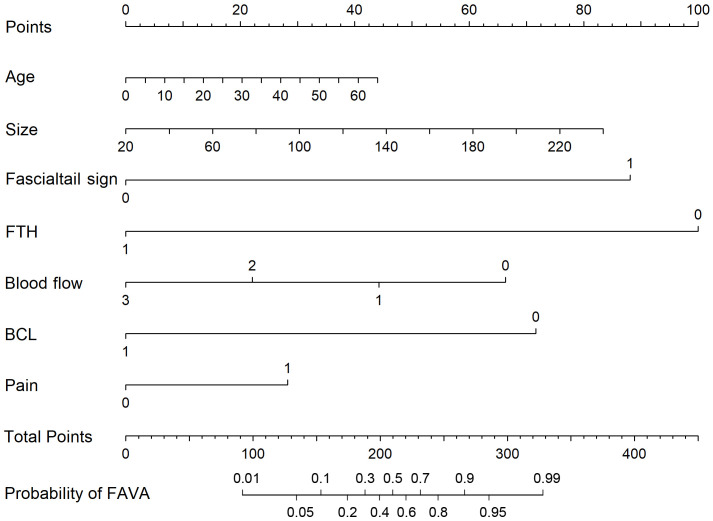
Diagnostic nomogram that incorporates meaningful variables together with the “Fascialtail sign” and “presence of blood capillaries that go straight within the lesion”. FTH, presence of fatty tissue-like hyperecho around the lesion; BCL, presence of blood capillaries that go straight within the lesion; Pain, pains and sensitivity to pressing; FAVA, fibro adipose vascular anomaly.

The results of a mono-factor analysis showed that the difference was statistically significant (*P*< 0.05) for variables including ‘age’, ‘lesion size’, ‘fascial tail sign’, ‘presence of fatty-tissue-like hyperecho around the lesion’, ‘blood flow’, ‘presence of straight blood capillaries within the lesion’ and ‘pain and sensitivity to pressing’, but it was not statistically significant for the other variables (see **Table 1**). A diagnostic nomogram that incorporated the meaningful variables was created using the R studio program (see **Figure 1**).

**Table 1:** Comparison of general clinical features and ultrasound signs between ICTH and FAVA patients.

Variable	Group		Statistics	*P*
	ICTH (N=20)	FAVA (N=45)		
Gender [cases(%)]			0.39	0.54
Male	9 (45.0)	24 (53.3)		
Female	11 (55.0)	21 (46.7)		
Age (years, $\bar{x}$ ±s)	8.55±8.06	20.00±13.99	-3.53	<0.001^a^
Lesion size(mm, $\bar{x}$ ±s)	53.40±26.30	86.98±44.78	-3.18	0.001^a^
Is the boundary sharply defined [cases (%)			1.68	0.203
No	9 (45.0)	28 (62.2)		
Yes	11 (55.0)	17 (37.8)		
"Fascialtail sign" [cases (%)]			21.11	<0.001
No	19 (95.0)	15 (33.3)		
Yes	1 (5.0)	30 (66.7)		
Presence of fatty tissue-like hyperecho around the lesion[cases (%)]			40.68	<0.001
No	2 (10.0)	41 (91.1)		
Yes	18 (90.0)	4 (8.9)		
Blood flow [cases (%)]			21.71	<0.001
0	0	1 (2.2)		
Level I	0	25 (55.6)		
Level II	8 (40.0)	12 (26.7)		
Level III	12 (60.0)	7 (15.6)		
Presence of blood capillaries that go straight within the lesion [cases (%)]			36.11	<0.001
No	6 (30.0)	45 (100.0)		
Yes	14 (70.0)	0 (0.0)		
Anatomical position [cases (%)]			0.067	0.796
Muscle only	19 (95.0)	42		
Involvement of extra-muscular	1 (5.0)	3		
Pain or sensitivity to pressing			8.825	0.003
No	12	10		
Yes	8	35		

a is for the independent sample t-test, and the rest is for the chi-square test.

ICTH, intramuscular capillary-type hemangioma; FAVA, fibro adipose vascular anomaly.

### Construction of multi-factor joint diagnostic model

The significant variables obtained from the mono-factor analysis were processed using a multi-factor logistic stepwise regression analysis (backward regression) for stepwise selection. The final variables selected only included ‘fascial tail sign’ (*P* = 0.013) and ‘presence of straight blood capillaries within the lesion’ (*P* = 0.001). The results showed that the finalized logistic model was statistically significant (see **Table 2**).

**Table 2:** Use of the binary logistic stepwise regression analysis to differentiate ICTH and FAVA according to ultrasonographic features .

Variable and constant	Regression coefficient	OR value	95% *CI*	*P* value
Fascialtail sign	2.889	17.975	1.823-177.194	0.013
Presence of blood capillaries that go straight within the lesion	-3.241	0.039	0.006-0.253	0.001
Constant	0.934	2.546		0.062

ICTH, intramuscular capillary-type hemangioma; FAVA, fibro adipose vascular anomaly.

As the two variables ‘fascial tail sign’ and ‘presence of straight blood capillaries within the lesion’ selected by the multi-factor logistic stepwise regression analysis had statistically significant regression coefficients, the following joint diagnostic equation could be constructed:

Lnodds = 0.934 + 2.889 × ‘fascial tail sign’ − 3.241 × ‘presence of straight blood capillaries within the lesion’ (see **Table 2**).

3 Comparison of diagnostic efficacy of the single and combined indicators.

**Figure 2:**
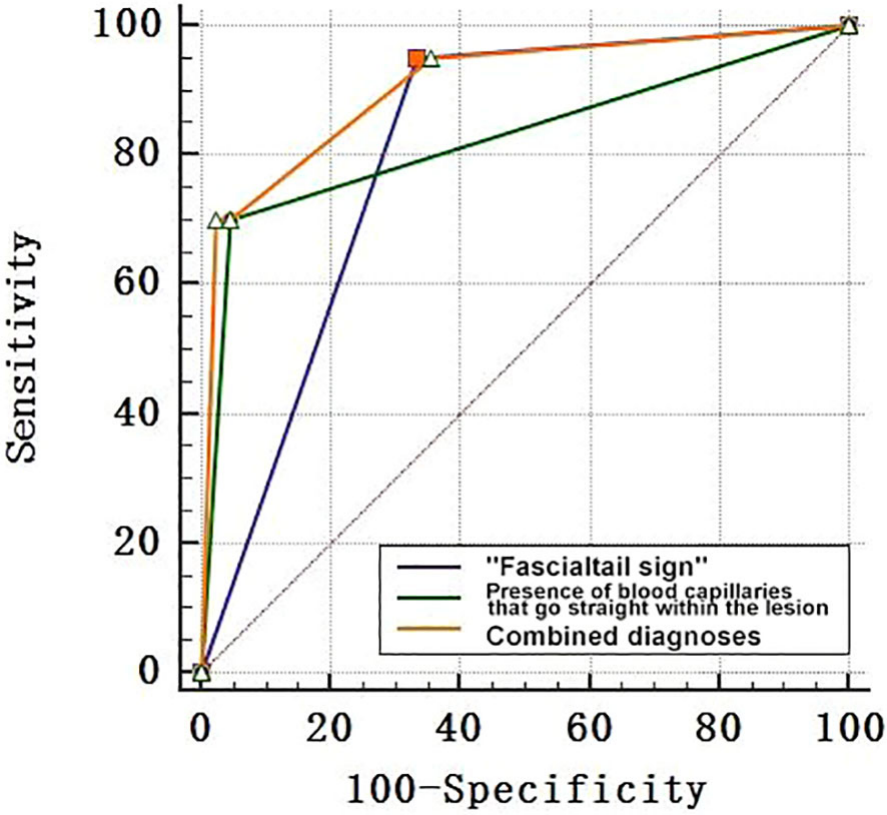
ROC curve of the joint diagnostic model. ROC curve, receiver operating characteristic curve.

**Figure 3:**
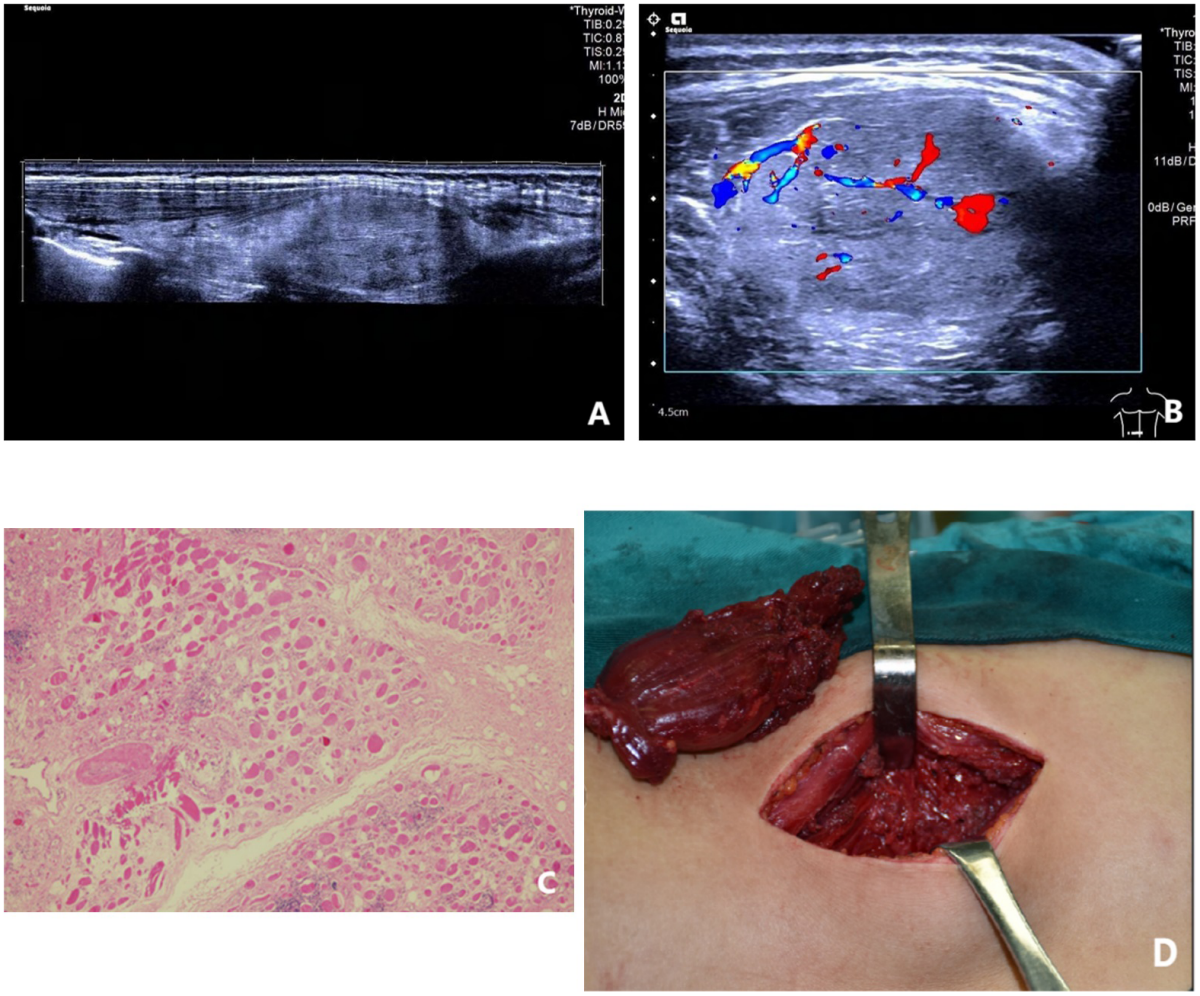
A female patient, 19 years old and suffering from intramuscular capillary-type hemangioma on the left back; **(A)** the panoramic imaging technique was used to longitudinally observe the lesion, and the results show a hypoechoic mass in the erector spinae muscle, with the fatty-tissue hyperecho around the lesion and normal strike of muscle fibers that might be seen indistinctly within the lesion; **(B)** CDFI suggested abundant blood flow signal and visibly straight strike of blood capillaries within the lesion; **(C)** the pathology showed that the new capillaries grew in an interspersed manner among the mature skeletal muscle fibers and could separate the skeletal muscle fibers. New capillaries were arranged in a “checkerboard form” and HE was magnified by small multiples; **(D)** during the operation, a dark red tumor with a soft texture was found to appear in the deep layer of the erector spinae muscle of the left lower back.

**Figure 4:**
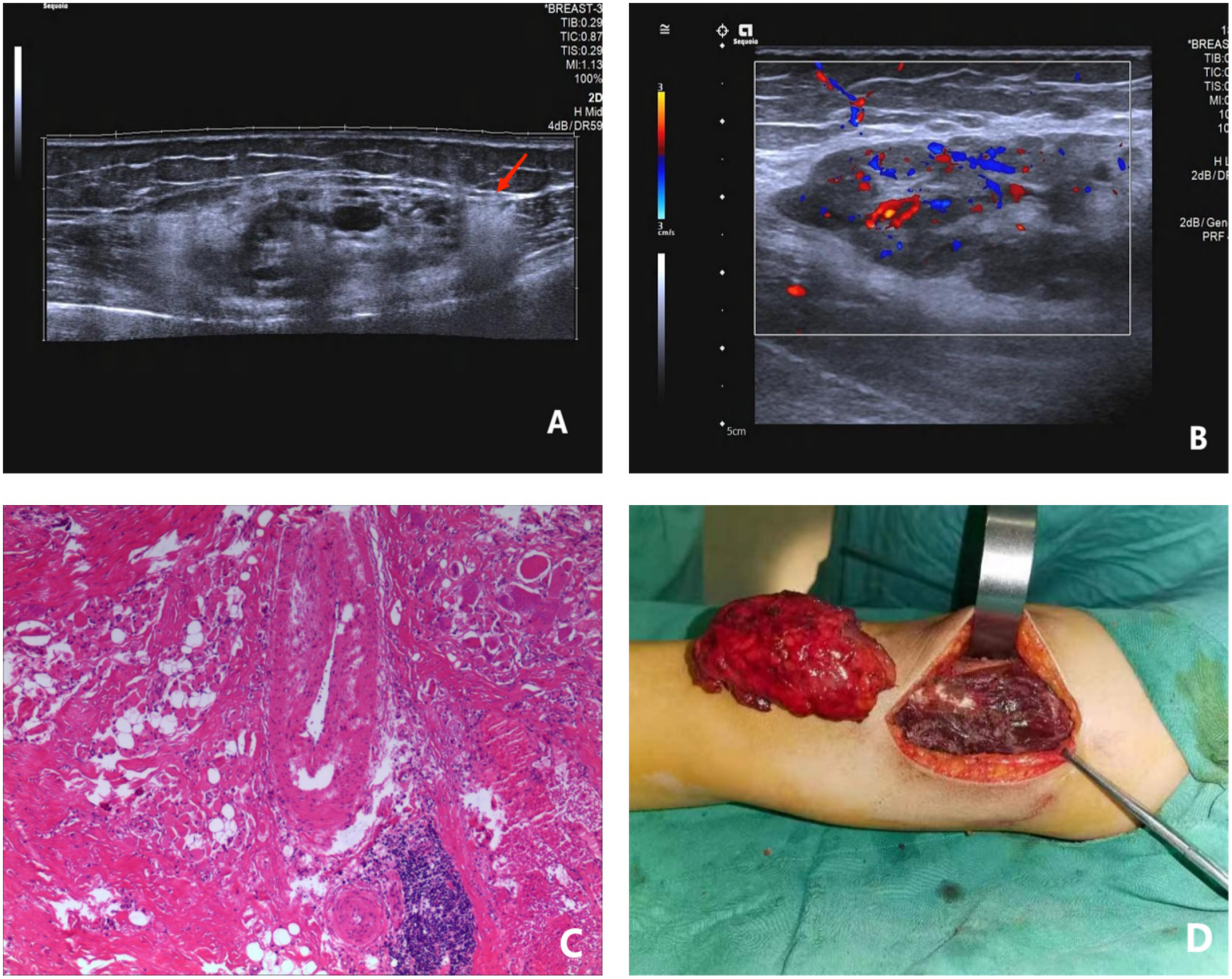
A female patient, 12 years old and suffering from intermuscular FAVA in the right thigh; **(A)** longitudinal 2D ultrasound image showed a mixed intermuscular solid mass with triangular hyperechoic “fascialtail sign” (red arrow) at the lower edge of the lesion; **(B)** through enlargement of local view of the lesion, CDFI suggested a small amount of blood flow signal within the lesion; **(C)** pathological findings showed existence of abnormally dilated vessels, fatty tissues and fibrous tissues in muscle cells. Fiber content could be found around the lesion, HE×100; **(D)** a bright red tumor with a tough texture was found during the operation in the deep layer of the right thigh muscle. FAVA, fibro adipose vascular anomaly.

Using combined indicators, the model’s diagnostic sensitivity was 70.0% (95% CI: 59.00–81.00), its specificity was 98.0% (95% CI: 94.7–100.0), its area under the ROC curve was 0.908, and the corresponding diagnostic cut-off value was 0.062. The comparison results of the ICTH/FAVA images showed that the single-indicator model (‘fascial tail sign’/’presence of straight blood capillaries within the lesion’) had high sensitivity and low specificity, while the joint diagnostic model that used combined indicators had low sensitivity and high specificity (**Table 3**, **Figures 2**–**4**).

**Table 3:** Comparison of efficacy between single-indicator and combined diagnoses.

Diagnostic indicator	Sensitivity	Specificity	Youden Index	AUC
Fascialtail sign	95.0 (90.0-100.0)	67.0 (56.0-78.0)	62.0 (50.0-74.0)	0.808 (0.701-0.915)
Presence of blood capillaries that go straight within the lesion”	96.0 (91.3-100.0)	70.0 (59.00-81.00)	66.0 (54.00-78.00)	0.850 (0.725-0.975)
Joint diagnostic model	70.0 (59.00-81.00)	98.0 (94.7-100.0)	68.0 (56.7-79.3)	0.950 (0.901-0.999)

AUC, Area Under the Curve.

## Discussion

Intramuscular capillary-type hemangioma and FAVA may overlap in terms of the age of onset and the pathogenic site. In the group with ICTH, the mean age of onset was 8.55 ± 8.06 years, which is younger than that of the group with FAVA. This is consistent with previous findings. The FAVA group had a larger lesion size than the ICTH group, with painless masses at the early stage despite clinical symptoms of progressive pain (11). It is difficult to distinguish between the two simply by clinical symptoms and lesion size. For this purpose, the use of ultrasonography to provide a basis for differential diagnosis has realistic research value.

Ultrasonography, as a testing method for non-invasive imaging, has been widely applied to evaluate the internal structure and blood supply of soft tissue masses in children. According to previous literature, ICTH ultrasonograms mainly show an inhomogeneous solid mass with well-defined borders within the muscle, which is surrounded by a fatty-tissue-like echo, with an inhomogeneous echo, free of a vessel-like echo and free of calcification or liquefaction. Colour Doppler flow imaging suggests a rich blood supply in the lesion (4). FAVA ultrasonograms mainly show the normal fibrous structure of muscle replaced by an inhomogeneous solid mass within muscular bundles or between muscles; this has a well-defined border, an inhomogeneous internal echo and echoless tortuous, malformed and dilated thin vasculature. Colour Doppler flow imaging suggests an unabundant blood supply in the lesion. The present study suggests that both are located within the skeletal muscle. However, in the former case, the muscle fibres are normal and uneroded in the lesion and are surrounded by a fatty-tissue-like hyperecho. This is consistent with the findings of previous literature (4). In the latter case, the boundary is clear, and the normal intramuscular muscle fibres that cannot be displayed in the image are replaced by fibres and fat mixed with deformed vascular structures. The inconspicuous fatty-tissue-like hyperecho around the lesion is replaced by a fascial tail sign at the top and bottom of the lesion (12), with a positive correlation with the symptoms of FAVA (regression coefficient: 2.889).

This study also revealed that compared with FAVA, ICTH has a richer blood supply, and there are straight capillaries within the lesion. Those symptoms are positively correlated with the diagnosis of ICTH (regression coefficient: −3.241). Relevant literature suggests that an ICTH tumor is mainly composed of proliferated capillary masses, and a large number of endothelial cell masses grow in a piece or lobular shape (interspersed/separated by mature skeletal muscle fibres) or partly in a checkerboard-like shape; the muscular vasa vasorum visible in lobular structures are increased in number at tumor margins and resemble muscular arterioles or venules (3, 10, 13, 14). This vessel acts as a feeding artery branch for the tumor and differs from the deformed tortuous dilated vessels inside FAVA lesions, which is conducive to the differentiation of the two diseases.

To improve diagnostic value, this study weighted the dominance ratio of the variables ‘fascial tail sign’ and ‘presence of straight blood capillaries within the lesion’ and established a joint diagnostic equation in which the area under the curve was significantly improved to 0.908, and the equation was proven to be more effective than the single-indicator equation. By detailing the real evaluation indicators, the method provides a new idea to effectively integrate multi-factorial information for the diagnosis of ICTH and can improve diagnostic specificity to a certain extent (98.0%).

The limitations of this study are as follows: (1) Because a retrospective study design was used, all findings need to be validated by prospective diagnostic tests. (2) Due to the small sample size, it is necessary to collect additional case data for further validation. (3) In this study, semi-quantitative criteria were applied to assess the blood flow within lesions. In the future, microangiography or ultrasonography can be utilized during prospective diagnostic tests for blood flow evaluation.

## Conclusion

In summary, this study effectively compared the ultrasonographic characteristics of ICTH and FAVA and systematically evaluated their practical application value in differential diagnosis. The results showed that the indicators ‘fascial tail sign’ and ‘presence of straight blood capillaries within the lesion’ can be used for differential diagnosis. The joint diagnostic equation established by weighting the above factors can effectively improve the diagnostic efficiency of ultrasonography and thus has high application potential.

## Data availability statement

The raw data supporting the conclusions of this article will be made available by the authors, without undue reservation.

## Ethics statement

The studies involving humans were approved by Ethics Committee of Henan Provincial People’s Hospital. The studies were conducted in accordance with the local legislation and institutional requirements. The participants provided their written informed consent to participate in this study. Written informed consent was obtained from the individuals or minors’ legal guardian/next of kin for the publication of any potentially identifiable images or data included in this article.

## Author contributions

W-JH: Conceptualization, Writing – original draft, Writing – review & editing. H-TL: Conceptualization, Writing – original draft, Writing – review & editing. Z-NF: Data curation, Writing – original draft, Writing – review & editing. Y-BG: Data curation, Writing – original draft, Writing – review & editing. X-NG: Data curation, Writing – original draft, Writing – review & editing. C-XD: Formal Analysis, Writing – original draft, Writing – review & editing. P-HF: Formal Analysis, Writing – original draft, Writing – review & editing. XY: Writing – original draft, Writing – review & editing. GW: Writing – original draft, Writing – review & editing.
